# The “Cairo Accord”- Towards the Eradication of RHD: An Update

**DOI:** 10.3389/fcvm.2021.690227

**Published:** 2021-07-02

**Authors:** Susy Kotit, David I. W. Phillips, Ahmed Afifi, Magdi Yacoub

**Affiliations:** ^1^Aswan Heart Centre, Aswan, Egypt; ^2^Developmental Origins of Health and Disease Division, University of Southampton, Southampton General Hospital, Southampton, United Kingdom; ^3^Heart Science Centre, National Heart and Lung Institute, Imperial College London, London, United Kingdom

**Keywords:** Cairo accord, neglected disease, eradication, vaccine, echocardiography, screening, rheumatic heart disease, RHD

## Abstract

Rheumatic heart disease (RHD) is the most common cause of acquired heart disease in children and young adults. It continues to be prevalent in many low- and middle-income countries where it causes significant morbidity and mortality. Following the 2017 Cairo conference “Rheumatic Heart Disease: from Molecules to the Global Community,” experts from 21 countries formulated an approach for addressing the problem of RHD: “The Cairo Accord on Rheumatic Heart Disease.” The Accord attempts to set policy priorities for the eradication of acute rheumatic fever (ARF) and RHD and builds on a recent series of policy initiatives and calls to action. We present an update on the recommendations of the Cairo Accord and discuss recent progress toward the eradication of RHD, including contributions from our own Aswan Rheumatic Heart Disease Registry (ARGI).

## Introduction

Rheumatic heart disease (RHD) is a late consequence of acute rheumatic fever (ARF) following group A streptococcal (GAS) infection (1). It is the most common cause of progressive acquired heart disease in children and young adults (2, 3). Although once common throughout the world, the disease burden is now almost entirely limited to low and middle income countries and the poor indigenous populations of some wealthy countries (3–5). RHD continues to be an important cause of mortality and disability and yet remains a neglected disease (6–8). While there is strong evidence linking RHD to poor socio-economic (9, 10) and environmental conditions (11, 12), the underlying pathogenesis, particularly the reasons for host susceptibility (13–18) and bacterial rheumatogenicity (19, 20), remain poorly understood.

**Figure 1:**
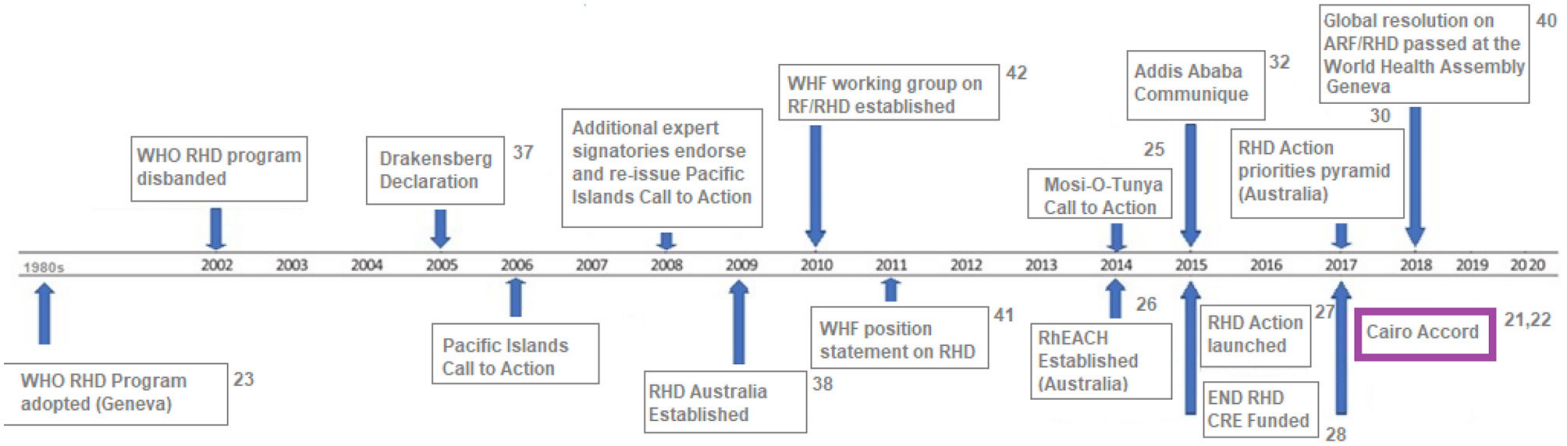
Global and regional RHD-specific policy activity and calls to action, 1980 – 2020 [adapted from (23)].

Developing appropriate strategies to deal with this problem remains a top priority in public health. In response, the Cairo Accord was formulated in 2017 following a meeting entitled “Rheumatic Heart Disease - From Molecules to The Community” (21, 22) and attempts to set criteria and priorities for addressing the problems related to RHD and achieving the eradication of RHD through different approaches. The Accord, in turn, builds and expands on a continuing series of initiatives around the world (Figure 1) (21, 24–41).

It stresses the need for a comprehensive systematic approach to the problem incorporating basic science, epidemiological, and clinical components. The recommendations include establishing standardized data collection procedures and databases for epidemiological studies; establishing clear echocardiographic procedures for diagnosis through research-driven studies; improving research into the interaction between and dependence on host genetics and pathogen source on the prevalence of RHD; the development of a vaccine; and improved engagement from international experts in valve surgery.

We here present an update of the areas enumerated by the Cairo Accord and discuss progress toward the eradication of RHD, including our own Aswan Rheumatic Heart Disease Registry (ARGI).

## The “Cairo Accord”: Update on the Recommendations

**1. “Call for obtaining more accurate data on the epidemiology and natural history of the disease by strengthening the existing databases, and by ensuring that different databases are capable of cross-communication and data exchange.”**

Generating accurate data on the epidemiology and natural history of RHD is a vital tool for both the prevention and control of the disease as well as for programme planning and advocacy activities. Compared with many other diseases there has been a lack of routine data on disease occurrence and hitherto much has depended on national mortality recording systems or hospital-based records. The past few years have, however, seen considerable development and strengthening of registries at global, national and hospital-based level.

### Global Databases

**Figure 2:**
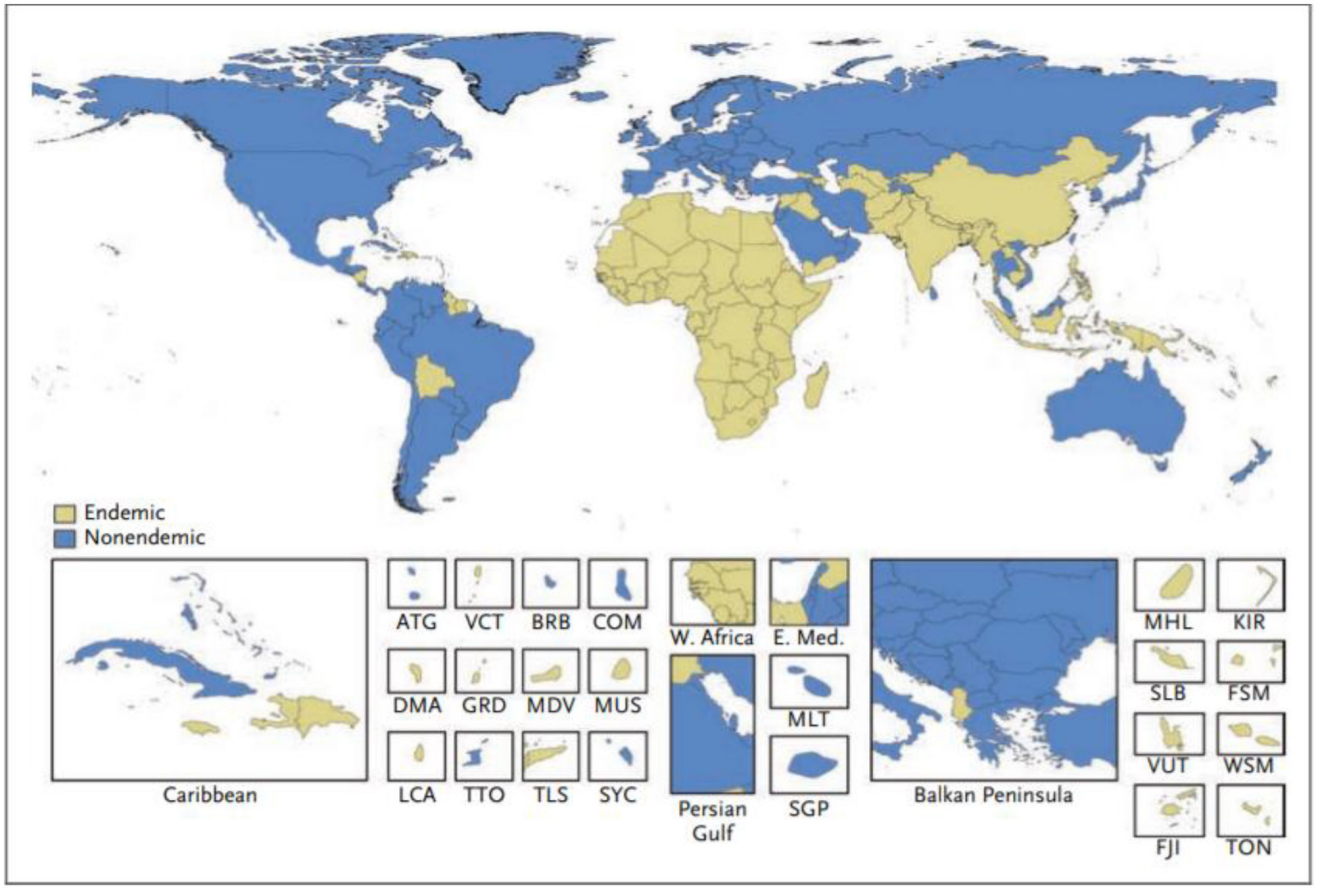
Classification of countries as having an endemic or non-endemic pattern of rheumatic heart disease. A country was classified as having an endemic pattern of disease if its estimated childhood mortality due to rheumatic heart disease was greater than 0.15 deaths per 100,000 population among children 5 to 9 years of age. ATG, Antigua and Barbuda; BRB, Barbados; COM, Comoros; DMA, Dominica; E. Med., Eastern Mediterranean region; FJI, Fiji; FSM, Federated States of Micronesia; GRD, Grenada; KIR, Kiribati; LCA, Saint Lucia; MDV, Maldives; MHL, Marshall Islands; MLT, Malta; MUS, Mauritius; SGP, Singapore; SLB, Solomon Islands; SYC, Seychelles; TLS, Timor-Leste; TON, Tonga; TTO, Trinidad and Tobago; VCT, Saint Vincent and the Grenadines; VUT, Vanuatu; W. Africa, West Africa; WSM, Samoa (42).

An encouraging number of studies now report estimates of the global prevalence of RHD. For example, Watkins et al. (42) combined multiple data sources with modeling techniques to evaluate global prevalence and mortality of RHD over a 25-year period. Although RHD has declined in most countries, it remains a major problem in sub-Saharan Africa, South Asia, and Oceania (Figure 2). During 2015, the authors estimate that globally there were 319,400 deaths and 33.4 million cases of RHD as well as the loss of 10.5 million disability-adjusted life-years (DALYs). However, the authors stress the continuing poor quality of data and misclassification of causes of death, which is a particular problem in low and middle-income countries (LMICs) where weak health systems are associated with a high prevalence of RHD.

Another welcome addition from the Institute for Health Metrics and Evaluation (IHME) is a review of “Rheumatic heart disease burden, trends, and inequalities in the Americas.” In this report they estimated age-adjusted incidence, mortality and DALYS in 37 counties throughout South America. In spite of the considerable drop in both incidence and mortality during the period of the study (1990-2017), there were marked regional inequalities related mainly to socioeconomic factors and facilities for RHD prevention and control (4). A particular problem in the estimation of the true burden of the disease is the use of surveys of schoolchildren in many population studies. Because RHD is linked with poverty, surveys carried out in schoolchildren will necessarily under-represent disease prevalence as poor children with RHD are less likely to attend school (5). Furthermore, because the peak incidence of RHD occurs after school age, school-based studies will necessarily underestimate the true burden of the disease.

### National Databases

**Figure 3:**
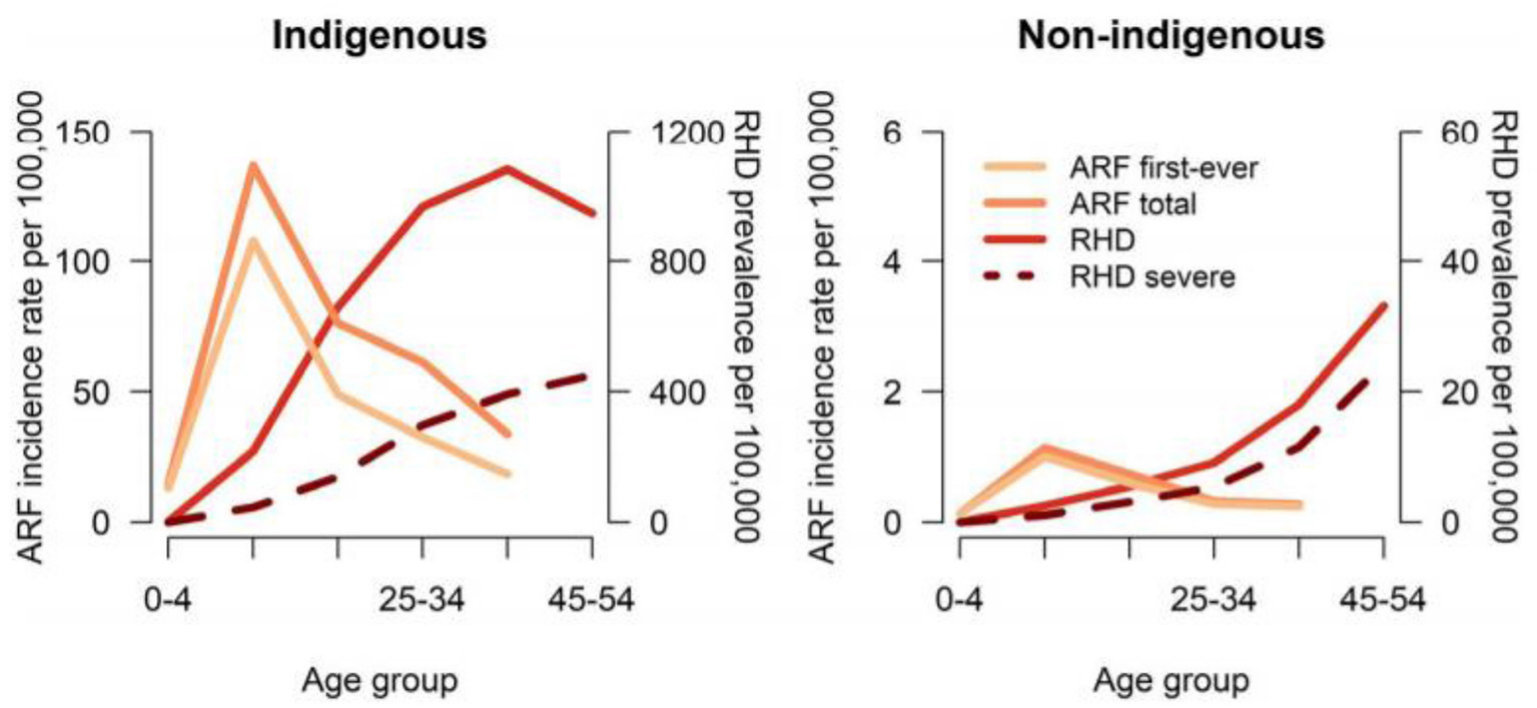
Age-specific incidence of ARF and prevalence of RHD in 5 Australian jurisdictions, by Indigenous status, 2015 to 2017. ARF total includes first-ever episodes of ARF plus ARF recurrences. Severe RHD includes RHD cases who were recorded as having been in heart failure, received at least 1 cardiac valvular intervention or were recorded on RHD register as being severe. ARF or RHD includes any live person with a history of either ARF or RHD. ARF, acute rheumatic fever; RHD, rheumatic heart disease (46).

**Figure 4:**
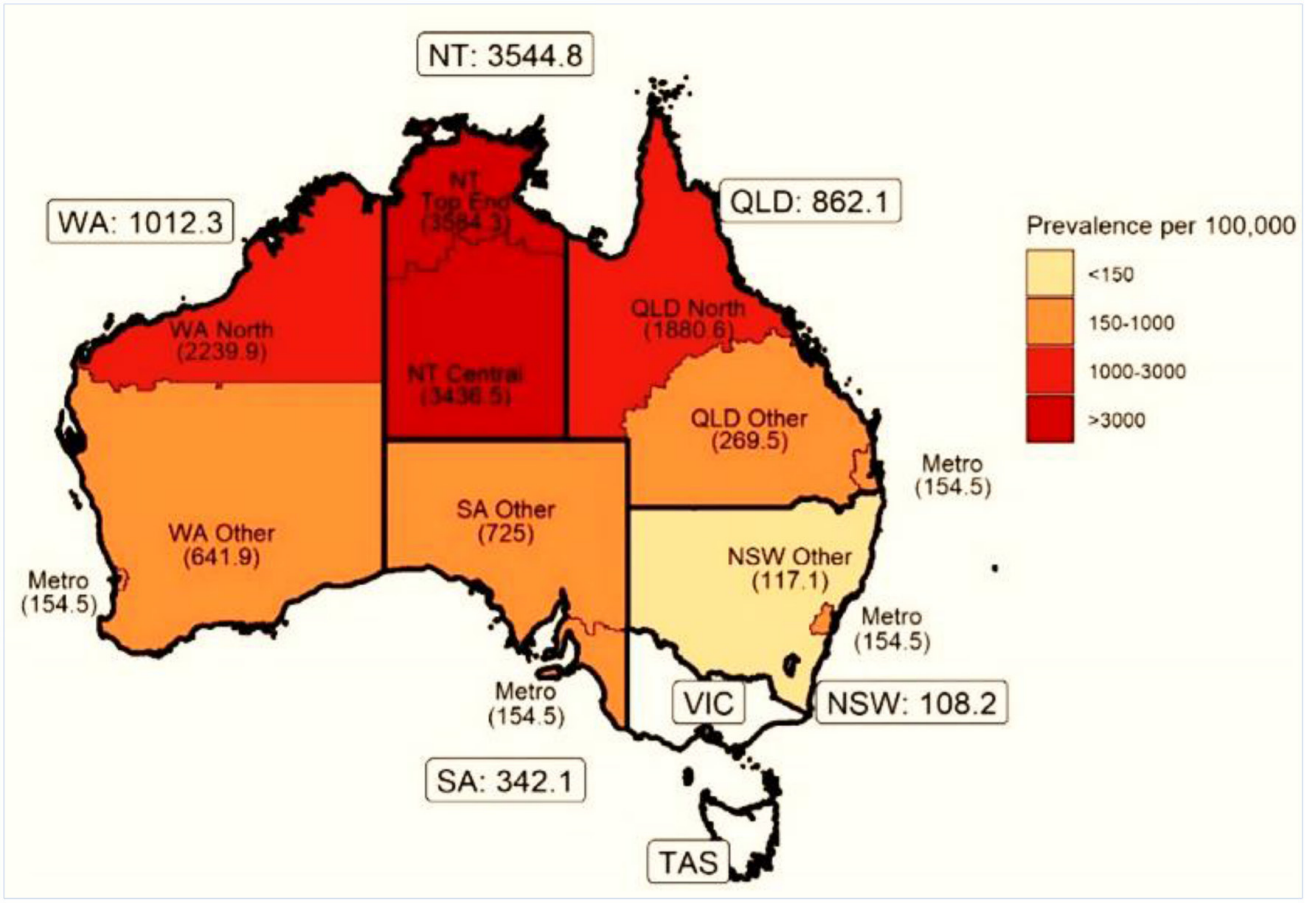
Age-standardized prevalence (per 100 000) of ARF or RHD in indigenous populations by state and residence. NSW, New South Wales; NT, Northern Territory; QLD, Queensland; SA, South Australia; TAS, Tasmania; VIC, Victoria; WA, Western Australia [modified from (46)].

Although progress in establishing national databases has been disappointingly slow, recent encouraging achievements have been the addition of RHD to the list of notifiable diseases in Queensland, Australia starting from September 2018 (43), and following a similar initiative in Western Australia in 2015 (44). Furthermore, a RHD action plan (2018-2021) has been developed to ensure that treatment and management is carried out in the best way possible, to aid preventive measures, and to assist the co-ordination of care (45). A multijurisdictional Australian study of acute rheumatic fever (ARF) and RHD using multiple person-linked longitudinal administrative data sources is a good example of what can be achieved. ARF and RHD cases were retrieved from linked ARF/RHD registers, inpatient hospitalizations and RHD-coded death registry data in 5 Australian jurisdictions (mid-2001–2018) to create a comprehensive database for characterizing the ARF/RHD patient population and estimating the burden of ARF and RHD. Using linked data provides more reliable estimates of disease burden as the linked dataset allows for a person's records to be followed across different data collections, compensating for the incompleteness of data from a single source. The longitudinal nature of the data allows an accurate estimation of disease onset and progression. Importantly this study suggested that previous data on disease burden had underestimated prevalence, while demonstrating substantial ethnic and subnational disparities in occurrence of the disease (see Figures 3, 4). This method has potential applicability elsewhere (46, 47) as a means of generating reliable data on the burden of disease.

### Hospital Based Databases

The increasing availability of both surgical and non-surgical treatment for RHD has underscored the need for the development of databases for the long-term follow-up of patients. Hospital-based registries have an important role in improving disease management, quality of care and clinical outcomes, monitoring anticoagulation, reducing loss to follow-up and the management of women with RHD during pregnancy in joint cardio-obstetric clinics. They can also be linked with national RHD registration systems. However, where they have been set up they do demonstrate the poor outcomes of current management. The Remedy study, a 2-year follow-up of a multicenter study of individuals with RHD from 14 LMICs in Africa and Asia, highlights the problems of treating the disease in resource-poor settings. Despite the young median age of patients (28 yr), the authors reported a 2-year case fatality rate of 16.9%, a mortality rate of 116.3/1000 patient-years in the first and a rate of 65.4/1000 patient-years in the second year (48). Other studies in sub-Saharan Africa report similar high mortality, for example the Mulago National Referral hospital in Uganda reported a 1-year mortality rate of 17.8% (49). In Aswan, a comprehensive database (ARGI) has been established since 2011 comprising over 1750 patients with RHD treated by medical, interventional or surgical procedures with plans to follow them up to improve outcomes.

Setting up RHD registries in resource poor countries, however, is a formidable challenge and progress is likely to be slow in the context of poorly-developed health systems already struggling to cope with both the traditional burden of communicable disease and the rising burden of non-communicable disease. One way forward may be the use of mobile and cloud technologies, which when combined with low-cost mobile phones and computer tablets and accessible data storage could be used to create electronic patient registers for RHD control programmes (50).

### Socioeconomic and Environmental Factors

The link between poverty and RHD is well-established (9, 51), and have led to various initiatives to address the disease (52–54). These include the provision of improved housing to reduce overcrowding and education. However, there is a clear need for a deeper investigation of the role of socioeconomic or environmental factors as they may identify specific poverty-linked determinants of RHD which may be amenable to public health intervention. Although ambitious, some possible approaches are emerging. Because ARF is a disease of childhood with a maximal incidence between 5 and 15 years of age, it is likely that any causative environmental factors operate during early childhood, a period of life which has tended to be neglected by most studies of RHD. Historical mortality studies carried out in the UK point to the operation of adverse environmental factors in early life. These studies demonstrated strong geographical associations between high rates of chest infection during infancy and RHD in later adult life. They were not explained by known risk factors such as domestic overcrowding (10). As air pollution is known to be strongly linked with chest infection during infancy, these associations raised the possibility that air pollution could be a factor in RHD susceptibility. The hypothesis was supported by the finding of strong geographical correlations between estimates of exposure to domestic air pollution in early childhood, based on coal consumption which was the major source of air pollution at that time, and adult mortality from chronic RHD (11, 12). Again these associations were not explained by overcrowding or related socio-economic factors. A role for air pollution is also mechanistically plausible as it affects epithelial integrity and has potent immunomodulatory effects, being implicated in the pathogenesis of other autoimmune diseases. If this association were valid, reducing domestic air pollution, which is widespread in many of the countries with high RHD prevalence and a major WHO public health priority, could be an important contributor to primary prevention of the disease.

**2. “Confine the use of echocardiographic screening programmes to research until further evidence regarding its impact on prognosis and cost-effectiveness is made available.”**

The past decade has seen considerable development of echocardiographic screening as a tool to identify subclinical or latent cases of RHD in the community. This has required agreement on standardized diagnostic criteria which have been developed by The World Heart Federation (WHF, 2012) (55). Several studies now show that echocardiographic screening is much more sensitive than auscultation, providing a detection rate up to 10 times greater than clinical examination alone. It is an important tool to estimate the burden of disease in populations and its use in community-based studies has provided more accurate data on disease prevalence, and important insights in uncovering the full spectrum of RHD (56, 57).

Controversial, however, is the use of echocardiography as a screening tool to identify cases of mild RHD that might be treated early to prevent clinical complications. A recent meta-analysis combined data from 12 studies following up patients with latent RHD. The authors' estimated prevalence progression for latent RHD (defined variously as worsening of the WHF grade or clinical severity of disease) was 5%/year, while in the fewer studies reporting on the progression of borderline RHD, progression was as low as 2%/year (58). Although these low rates do call into question the use of echocardiography for screening, there is clearly a need for longer term studies of the natural history of latent RHD and for identifying clinical predictors of disease progression.

The major disadvantage of echocardiography is the need for expensive equipment and a high degree of training, which is unlikely to be accessible outside specialist referral hospitals unless simpler screening techniques can be developed. Recently developed possibilities include the single parasternal-long-axis-view-sweep of the heart (SPLASH), which has been shown to be highly sensitive and specific with the potential to improve significantly the efficiency of RHD screening (59), and the use of cheaper, hand-held echo devices as screening tools. Nevertheless, the relatively high cost and complexity of screening have to be weighed against mitigating the risk of complications or mortality from RHD in the context of other demands on health services.

**3. “Enhance and coordinate research efforts on the genetics of rheumatogenic streptococcal strains and affected patients. The influence of ethnicity and epigenetics should be included in future studies.”**

### Rheumatogenic Streptococcal Strains

**Figure 5:**
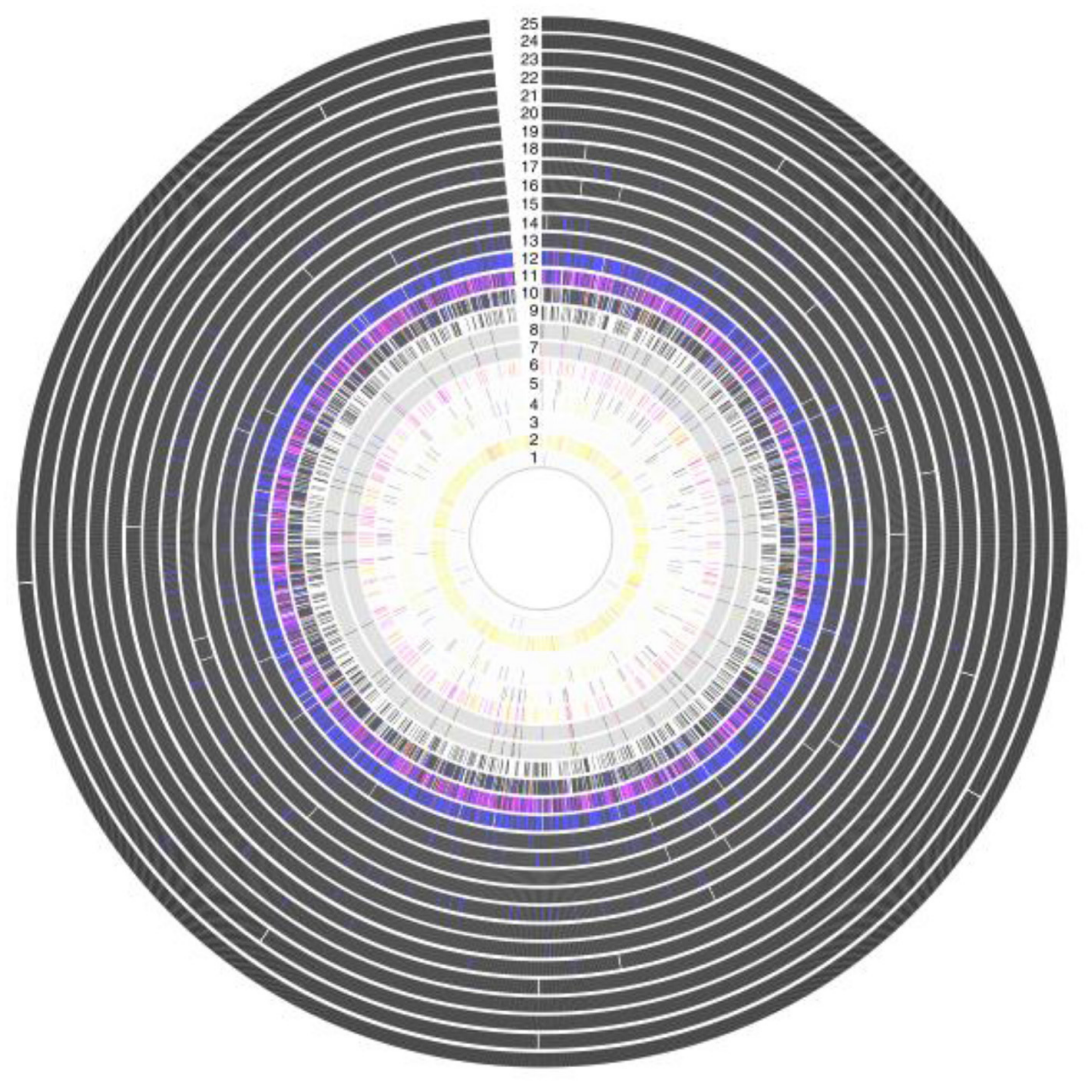
Antigenic variation within vaccine targets, showing a high grade of variation in Amino acid sequence within 25 protein antigens. Each ring represents a single antigen with protein similarity color coded according to pairwise BLASTp similarity: black (>98%); blue (95–98%); red (90–95%); pink (80–90%); yellow (70–80%); gray (<70%); and white (protein absence). Rings correspond to: (1) R28; (2) Sfb1; (3) Spa; (4) SfbII;(5) FbaA; (6) SpeA; (7) M1 (whole protein); (8) M1 (180-bp N terminal); (9) SpeC; (10) Sse; (11) Sib35; (12) ScpA; (13) SpyCEP; (14) PulA; (15) SLO; (16) Shr; (17) OppA; (18) SpeB; (19) Fbp54; (20) SpyAD; (21) Spy0651; (22) Spy0762;(23) Spy0942; (24) ADI; and (25) TF (19).

**Figure 6:**
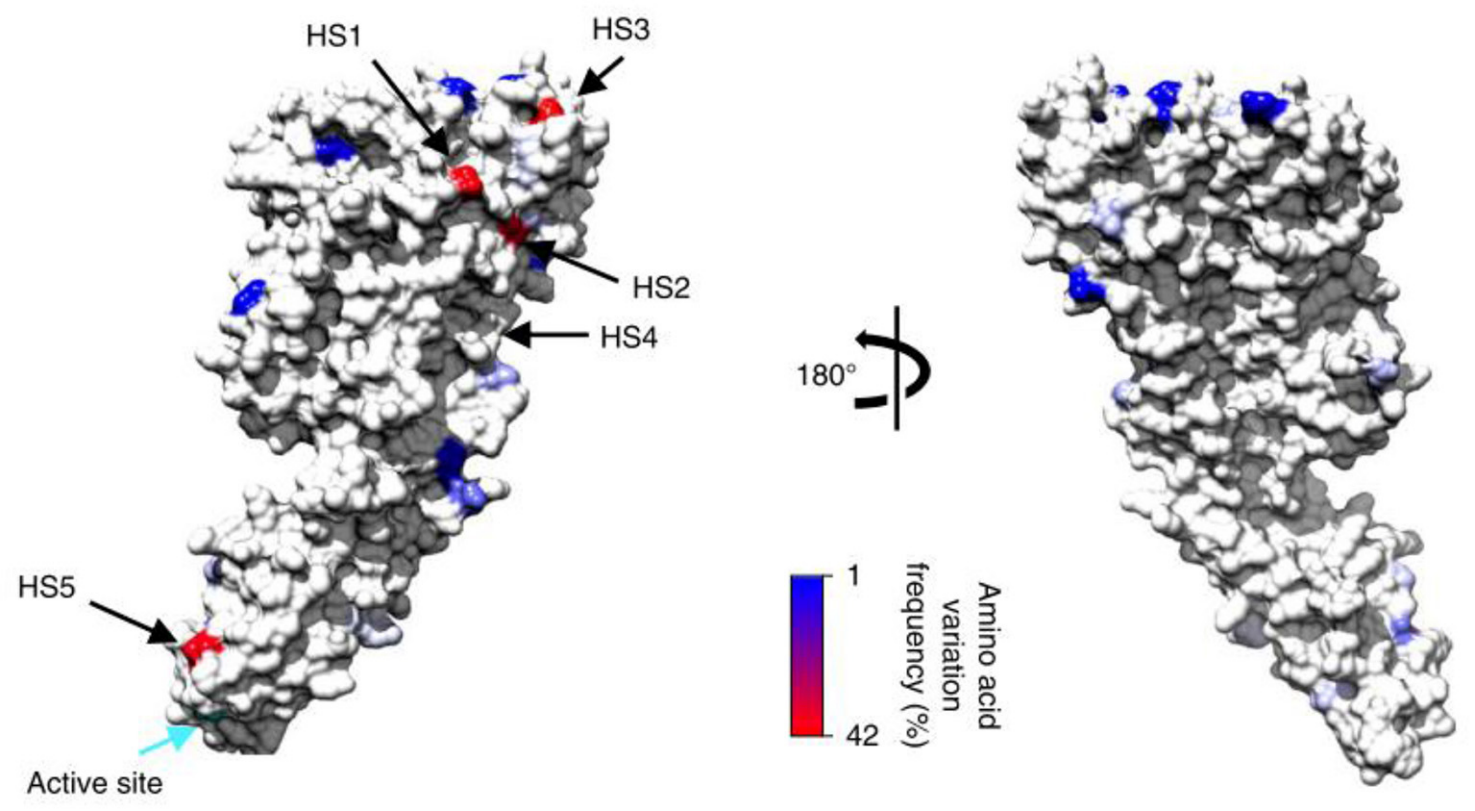
Modeled consensus amino acid sequence and population-derived polymorphisms of the mature streptolysin O protein onto the corresponding crystal structures based on identified polymorphism locations and polymorphism frequencies within GAS genomes for the streptolysin O. Plotted against each structure is the amino acid variation frequency within GAS genomes, as represented in the color gradient from 1% (blue) to 42% variable (red); invariant sites are colored light gray. The positions of the top five most variable surface hot spots (HS) are annotated. Active sites for each enzyme are also indicated (cyan arrow) (19).

Development of a streptococcal vaccine for the prevention of RHD has been hampered by the extensive global genomic heterogeneity of group A streptococcal (GAS) bacterial populations. This is driven by “homologous recombination and overlaid with high levels of accessory gene plasticity” (Figures 5, 6) (19). It is now clear that the concept of certain strains having enhanced rheumatogenic potential should be extended to include RHD caused by strains apart from those classically described. Recent studies show that there are still significant gaps in our understanding of the pathogenesis of rheumatic fever pathogenesis which will need to be addressed in order to develop a viable GAS vaccine (20). perhaps using reverse vaccinology as outlined later in this review (60).

### Host Genetic Susceptibility

Although, twin and family studies have long suggested that RHD might have a heritable component, until recently the evidence for this was limited. The search for susceptibility genes has been greatly enhanced by the advent of genome-wide association studies (GWAS) through which a very large number of variants can be tested for associations with RHD. A GWAS carried out on aboriginal Australians has provided evidence that variation at the HLA class 2 (DQA1-DQB1) locus is a major risk factor for RHD. The authors showed that human myosin cross-reactive epitopes of rheumatogenic streptococci were able to bind with higher affinity to the DQA1/DQB1 alpha/beta dimers of the risk haplotypes than in protective haplotypes (13). In contrast, a GWAS of New Caledonian and Fijian populations showed that the immunoglobulin heavy chain locus was significantly associated with RHD; (14, 15) the reasons for the differences are unclear.

Circulating exosomes and their cargoes, including mRNA and long non-coding RNA (lncRNA), are now thought to play an essential role in many cardiovascular diseases. Although not yet implicated in the pathogenesis of RHD, the first transcriptome analysis has recently identified differentially expressed lncRNAs and mRNAs in circulating exosomes from RHD patients which may be a route for identifying novel potential biomarkers and therapeutic targets (16).

MicroRNAs (miRNA) are fundamental for normal development, differentiation and growth control and are now implicated in many diseases. They are believed to be involved in valvular heart disease (VHD) related pathways including cell cycle control, inflammation and fibrosis, and are possible diagnostic and prognostic biomarkers for patients as well as therapeutic targets (16). Next generation sequencing of miRNAs has shown that the interleukin 1β and interleukin 1 receptor 1 might be involved in RHD. Two miRNAs, hsa-miR-205-3p and hsa-miR-3909 and their target genes IL-1β and IL1R1, appear to be specifically involved in disease progression, suggesting a potential augmentation of the IL1 pathway. However, more studies are needed on miRNA sequencing and quantitative reverse transcription–PCR analysis of miRNA expression levels (17). In another study of RHD, miR-1183 was demonstrated to be differentially expressed showing that significantly higher expression levels of miR-1183 through regulation of the anti-apoptotic protein, BCL-2, might affect myocardial apoptosis and remodeling (18).

#### Animal Models

**Figure 7:**
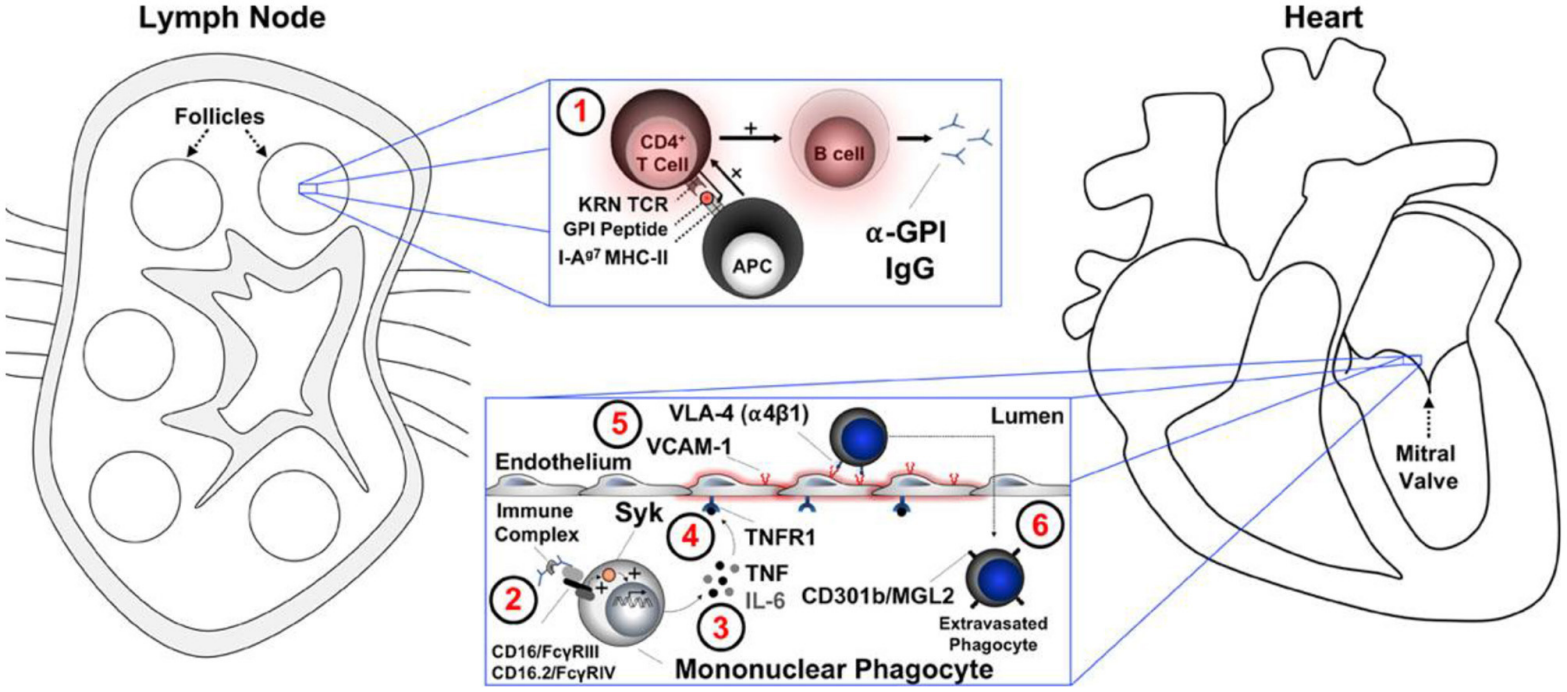
A summary working model for the initiation of cardiac valve inflammation and fibrosis in K/B.g7 mice. Anti–glucose-6-phosphate isomerase (α-GPI) IgG autoantibodies produced systemically in the secondary lymphoid tissue (spleen and lymph nodes) (1) activate valve-infiltrating mononuclear phagocytes (MNPs) in a spleen tyrosine kinase (Syk)–dependent process (2) resulting in local production of TNF and IL-6 from CX3CR1+CD301b/MGL2+ MNPs. Tumor necrosis factor (TNF) receptor−1 (TNFR1)–mediated activation of the valve stroma (3) promotes vascular cell adhesion molecule−1 (VCAM-1) upregulation (4) and subsequent recruitment of additional circulating CX3CR1+ MNPs via very late antigen−4 (VLA-4, α4β1 integrin) in a feed-forward process (5). Interstitial MNPs assume a tissue-reparative phenotype characterized by expression of CD301b/MGL2, resistin-like molecule-α, and arginase-1 (6). APC indicates antigen-presenting cell (e.g., dendritic cell); FcγR, Fc receptor; I-Ag7 MHC-II, major histocompatibility complex–II from the non-obese diabetic mouse strain; IgG, immunoglobulin G; IL-6, interleukin-6; KRN TCR, transgenic T-cell receptor; and MGL2, macrophage galactose N-acetylgalactosamine-specific lectin 2 (49).

Animal models of autoimmune cardiac valve inflammation and fibrosis associated with arthritis have shown that T-cell receptor transgenic mice spontaneously develop systemic autoantibody-associated autoimmunity, leading to fully penetrant fibroinflammatory mitral valve disease (MVD) and arthritis. The key drivers of autoimmune MVD in these K/B.g7 mice are also present in humans. Key inflammatory molecules that drive MVD in this model were Syk, TNF, interleukin-6, very late antigen−4, and vascular cell adhesion molecule– are shown in Figure 7 (61, 62).

**4. “Enhance and coordinate global efforts to produce a vaccine. Strategies to accelerate the production of an effective vaccine (e.g., reverse vaccinology) should be explored and utilized.”**

**Figure 8:**
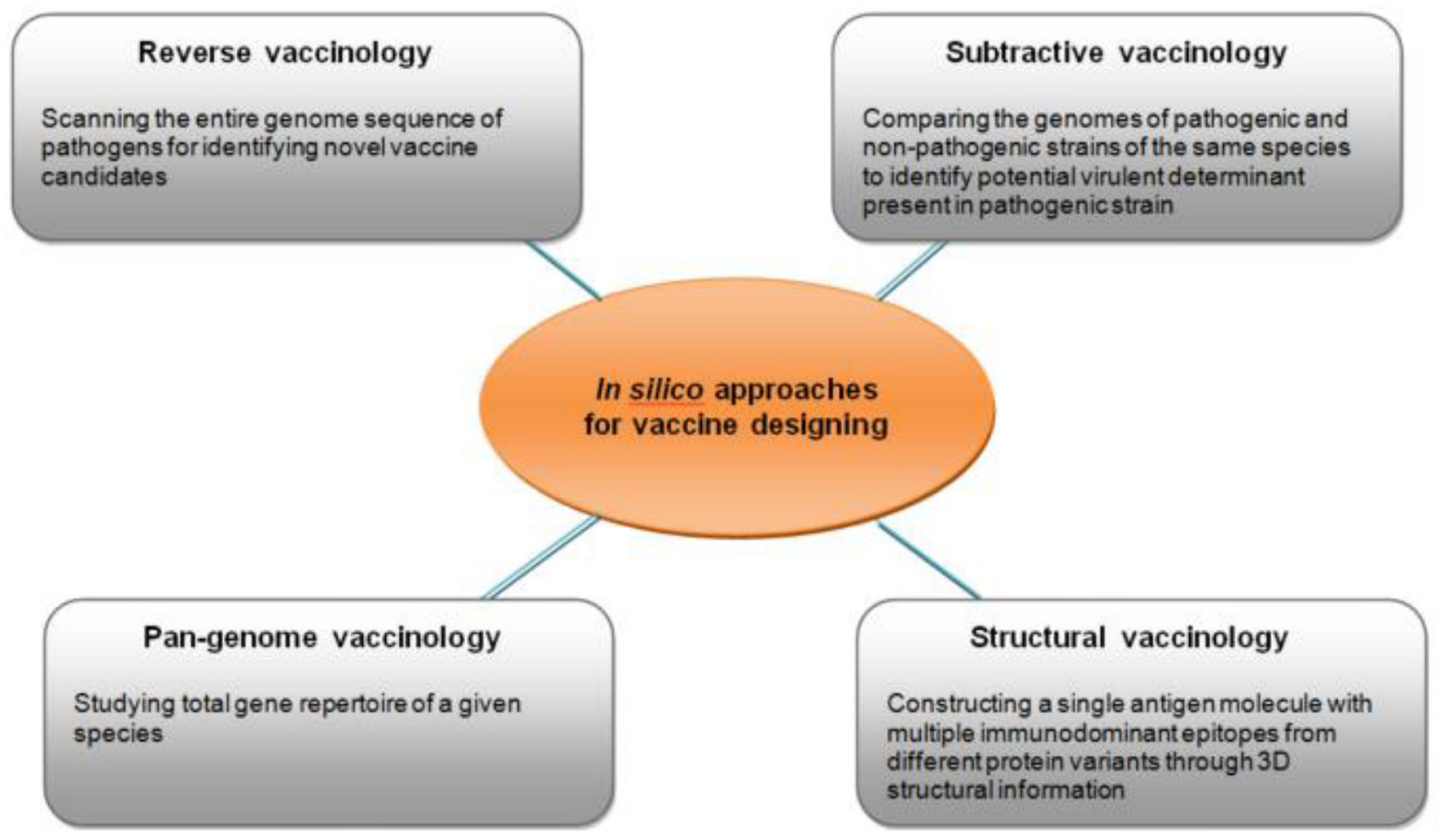
Different types of *in silico* approaches for identifying potential targets for vaccine development (60).

Gandhi and colleagues reviewed the potential vaccine targets in S. pyogenes and possible *in silico* approaches in developing a RHD vaccine (Figure 8) (60). This included reverse, subtractive and pan-genome vaccinology.

Work carried out in the Australian Northern Territories, has led to the development of a possible S. pyogenes “30mer” vaccine which is composed of 30 pharyngitis-associated type-specific antigens from the S. pyogenes M protein. The authors note that the effectiveness of the 30mer vaccine is “dependent on emm type cross protection in humans, which cannot currently be estimated with any certainty. If there is cross protection in accordance with cross opsonization data, then the 30mer vaccine could be reasonably predicted to provide good protection against all emm clusters except D1-5, and possibly variants, which together comprise 53.5% of the known samples. Therefore, a potential strategy would be to combine the 30mer vaccine with a vaccine(s) specifically targeting emm cluster D1-5, and possibly emm55. Accordingly, determination of the correspondence between cross opsonization data and clinical efficacy is critical for defining future directions prior to the consideration of testing” (63).

A randomized clinical trial in Queensland, using a novel acetylated peptide-protein conjugate vaccine candidate MJ8VAX (J8-DT) has been tested in humans and shown safety and immunogenicity with an increase in vaccine-specific antibodies which decreased over time. The vaccine was delivered intramuscularly to healthy adults. No serious adverse events were reported over 12 months but further investigations and changes in the formulation of the vaccine candidate will be required to evaluate the possibility for enhancing immunogenicity and assessing the number of doses needed for protection against GAS infection (64).

**5. “Develop biomarkers for early diagnosis and follow up of disease progression.”**

Levels of streptococcal antibody (antistreptolysin O, ASO), and/or antideoxyribonuclease B, ADB, titers), required as evidence of a preceding group A streptococcal infection appear to vary by population (65). However, neither ASO nor ADB antibody responses are sensitive nor specific enough to arbitrate whether or not there has been recent GAS infection. Recently, a novel antigen (SpnA) showed improved ability to detect a previous GAS exposure in ARF. Its sensitivity was 88% compared with 75% for streptolysin-O and 56% for DNaseB. Furthermore, SpnA can be combined with anti-streptolysin-O and anti-DNaseB in a multiplex single cytometric immunoassay to enhance efficiency and accuracy of ARF diagnosis (66).

A recent study in an RHD endemic area suggests that rapid nucleic acid molecular testing could detect GAS in the pharynx weeks after an infection. This could allow a higher detection rate of GAS than currently available throat swab cultures and improve management and antibiotic use in high-prevalence communities. However, further work will be required to explore apparent cross-reactivity with non-group A streptococcal strains, and the ability to detect nonviable GAS persisting after acute infection in patients with poststreptococcal syndromes (67).

Because levels of circulating inflammatory cytokines are associated the severity of RHD, measurements of cytokines in RHD patients may have potential as prognostic markers and provide a means for risk stratification. Recent evidence suggests that the co-regulated expression of IL-6 and TNF-α is associated with a worse clinical presentation and severe valve dysfunction, whereas IL-4 and IL-10 predict subsequent adverse outcomes (68).

**6. “Provision of high-quality penicillin to affected areas – for both primary and secondary prevention – continues to be an important priority. In parallel, longitudinal studies that provide robust evidence for the benefit of secondary prophylaxis on disease progression should be performed.”**

Penicillin has been the mainstay for the prevention of recurrences of rheumatic fever (secondary prophylaxis) as well as for treatment of symptomatic GAS infections (primary prevention). However, it must be recognized that disappearance of rheumatic heart disease from the developed world has led to neglect of the supply, manufacture, and accessibility of penicillin together with a lack of research into its effective dose and regimen and the cost benefits of different types of prophylaxis. Accordingly, clinical trials are sparse and current practice is based on historical trials carried out in a manner which today would not be considered acceptable.

Although there is good evidence that secondary prophylaxis with intramuscular (IM) penicillin is both effective and cost-effective at the community level, pharmacokinetic studies of benzathine benzylpenicillin G (BPG) administration in children or adolescents with RHD has shown that concentrations of BPG between injections are largely insufficient (69). Furthermore, while recurrent ARF most commonly occurs in the context of missed or late penicillin doses, a small number of individuals experience ARF recurrence despite acceptable compliance. Risk factors might include environmental factors, host immune factors, penicillin dosing, and pharmacokinetic considerations (70), but currently available data have not identified ways of predicting which individuals are most at risk. Poor compliance is a continuing problem, as demonstrated by a recent study funded by the Egyptian Ministry of health, WHO, WHF, and the African Union of 17050 individuals, reporting that 61.7% were non-compliant to the bi-weekly regimen of long-acting penicillin prophylaxis, thereby compromising efforts for the prevention of disease complications (71).

**Figure 9:**
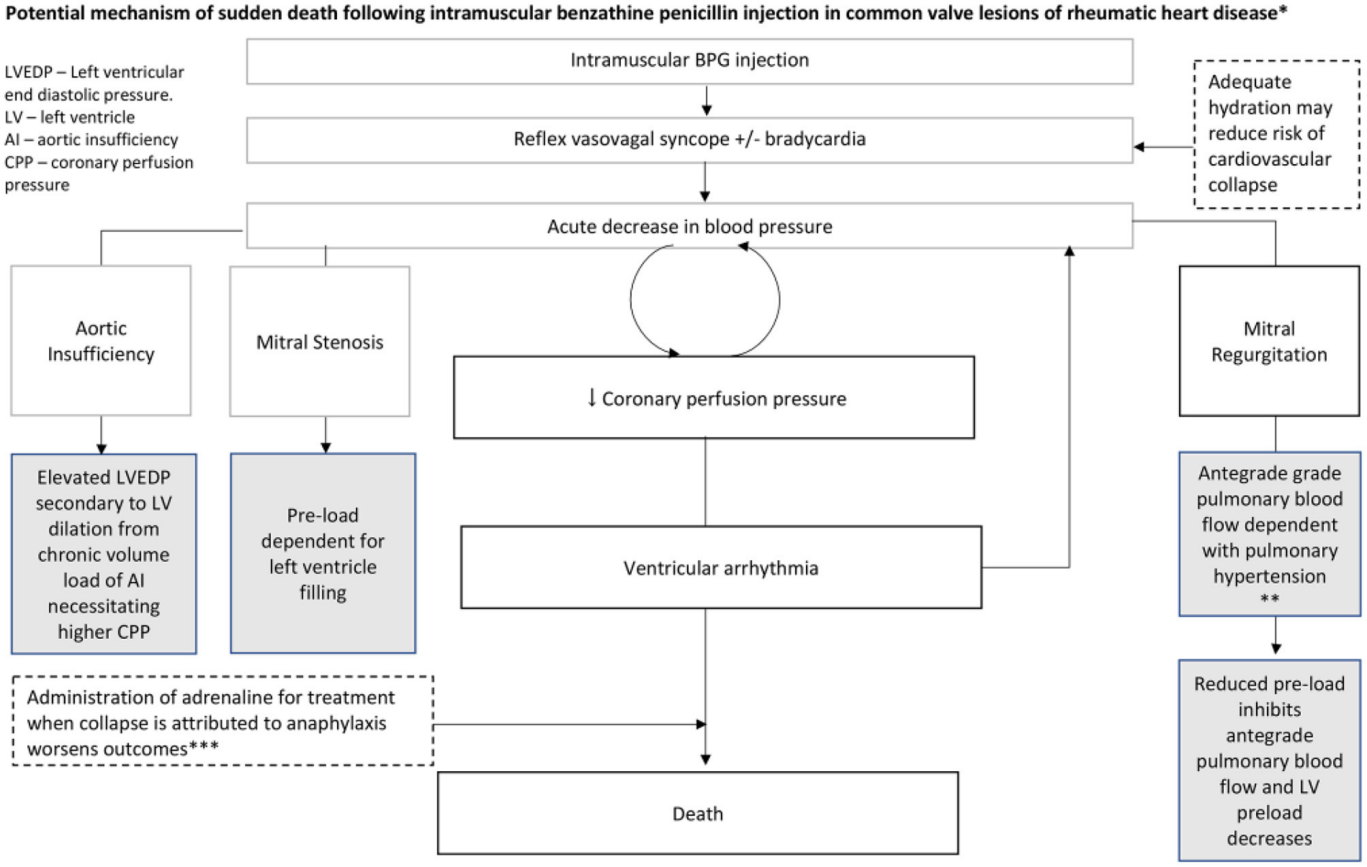
Potential mechanism of sudden death following intramuscular benzathine penicillin injection in common valve lesions of RHD. *Gray boxes reflect contributory or predisposing characteristics of valve lesions. Dashed boxes indicate other potential contributing/mitigating factors. *Plausible mechanism in this case series based on: very rapid onset, no respiratory or skin symptoms and no positive response to epinephrine. BPG, benzathine penicillin G (72).

Another problem with penicillin injections have been reports of fatalities. These do not appear to be related to anaphylaxis but might be due to production problems, substances used in manufacture, or the administration technique of BPG, although most are probably related to the underlying cardiac disease which makes individuals sensitive to reflex vasovagal syncope (Figure 9) (72).

We clearly need to know more about the pharmacokinetic/pharmacodynamic relationships between BPG administration and clinical outcomes. Specifically, there is a need for insights into the pharmacokinetic and pharmacodynamic characteristics of BPG and the formulation of improved secondary prophylaxis strategies and regimens (69, 70, 73). Currently, there is an ongoing randomized controlled trial to determine the impact of BPG prophylaxis in children with latent rheumatic heart disease (GOAL trial) (2018-2021). The purpose is to see whether secondary prophylaxis with 4-weekly injectable BPG improves outcomes for children aged 5 to 17 years in Northern Uganda diagnosed with latent RHD (74).

Biodegradable polymer matrices have been investigated as a way of reducing administration frequency and injection pain while improving adherence. Two different approaches have been evaluated, namely a parenteral injectable depot and monolithic surgical implant. The use of highly degradable poly(lactic acid) (PLA) and poly(lactic-co-glycolic) (PLGA)- based polymers were successful as measured by degradation rate and the release of contents. However, the acidic by-products of the degradation of PLA-based polymers also resulted in substantial penicillin degradation. Polycaprolactone (PCL) was studied as an alternative release matrix and although having favorable release behavior, it required an unacceptably large implant for the delivery of the required dose of drug. As yet, biodegradable PLGA-type systems do not appear to be suitable for the development of sustained release BPG treatments, and alternative approaches to degradable polymer systems are required (75).

Finally, although primary prevention has to be an important goal, there is still a lack of evidence that systematic screening and treatment of sore throats is cost effective (76). The widespread use of penicillin is difficult for resource-poor countries and the current political pressure to limit the use of antibiotics and prevent the emergence of resistance reinforces the need to identify alternative strategies.

**7. “Conduct studies that determine the potential value of anti-inflammatory/immunosuppressive therapy after acute rheumatic fever. Other important areas where evidence is needed include optimal stroke prevention strategies in patients with atrial fibrillation and/or mitral stenosis, and pharmacological management of those with heart failure.”**

Progress in this area has been modest. However, recent work has shown that the immune response to GAS in peripheral blood mononuclear cells is characterized by a dysregulated interleukin-1β-granulocyte-macrophage colony-stimulating factor (GM-CSF) cytokine axis, whereby persistent interleukin-1β production is coupled to overproduction of GM-CSF and selective expansion of CXCR3+CCR4–CCR6– CD4 T cells (66). Because hydroxychloroquine is a well-established and safe treatment for autoimmune diseases such as rheumatoid arthritis where GM-CSF plays a pivotal role, it is possible that this drug could be repurposed to reduce the risk of RHD after ARF (77).

**8. “Accelerate the development of regional Centers of Excellence equipped with both physical and human resources to deal with prevention and treatment of the disease. Linking these centers into various regional and global research networks will significantly enhance their performance and impact.”**

Sadly, the urgent need for development of regional Centers of Excellence equipped with both physical and human resources to deal with prevention and treatment of the disease remains unmet. Moreover, RHD research receives very little global funding despite its substantial disease burden. A study based on the Global Burden of Disease Study (78) with funding from the G-FINDER database (79), shows that across the range of evaluated diseases, RHD receives the least funding relative to its importance (80). Collaboration and the formation of multidisciplinary partnerships are essential in the fight against RHD and will need to involve both northern and southern partners.

A study performed by the Egyptian Ministry of health, also emphasizes the common occurrence of misdiagnosis and inappropriate primary prevention (71). Even the current International Classification of Disease (ICD-10) has limitations in its use for registering RHD (81). Survey work in recent years has suggested that RHD is widespread throughout both rural and urban areas of sub-Saharan Africa and other resource-poor countries. Here decentralization of integrated care is key and it seems feasible and practicable decentralize follow-up of patients with RHD to non-communicable disease clinics (82). Initiatives such as the PEN-plus partnership aim at establishing training sites and national operational plans to address the problems of managing diseases such as RHD in resource-limited situations (83). Finally, the COVID-19 pandemic presents both direct and indirect increased risks for people living with RHD. The COVID-19 pandemic is a significant challenge causing interruptions in secondary prophylaxis and reducing access to care, with potentially severe consequences. The pandemic is also likely to cause delay in surgical and catheter-based intervention as well as progress in RHD research, advocacy and country programmes (84).

**9. “Maximize the use of valve repair through educational programmes and exchange of expertise.”**

**Figure 10:**
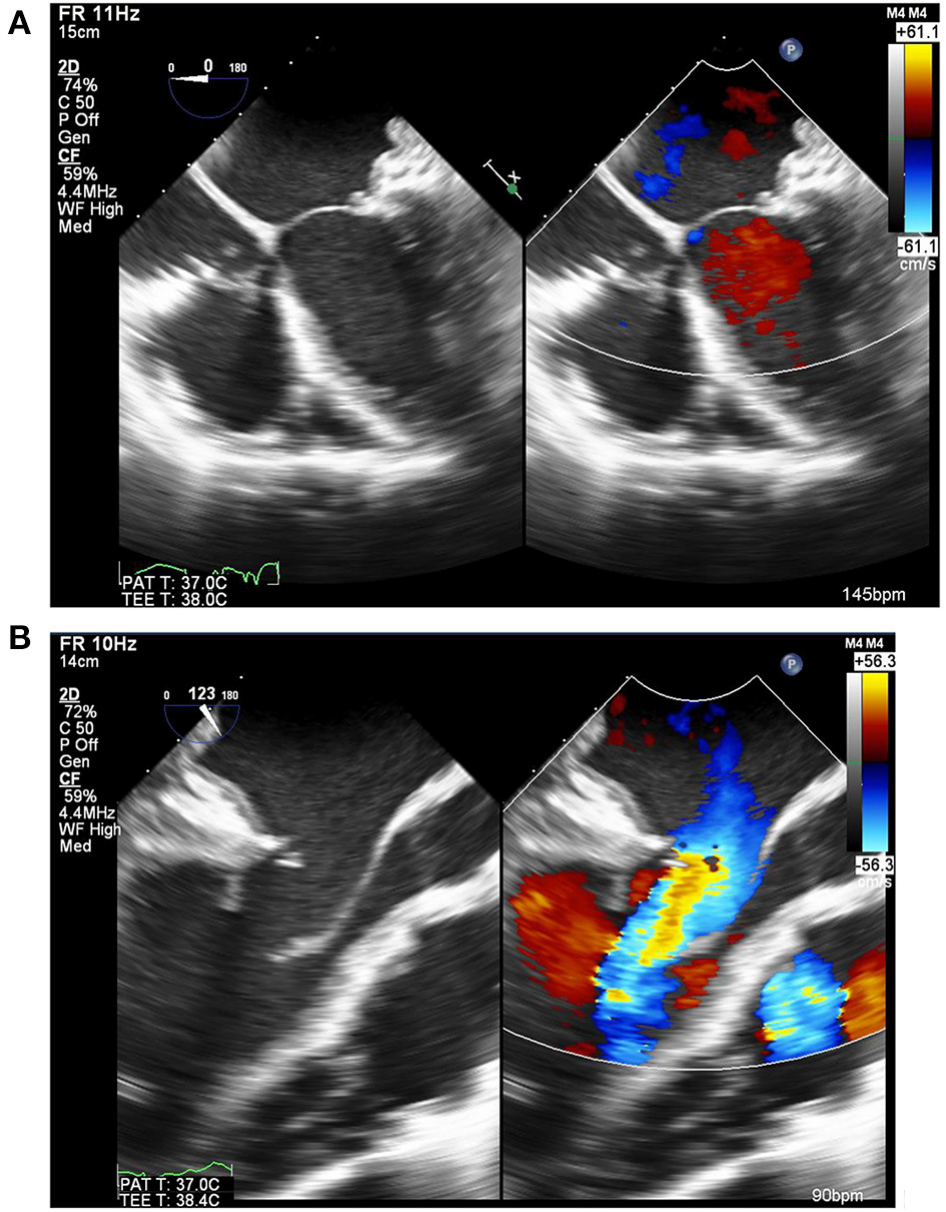
Echocardiogram of the mitral valve after repair by peeling of the anterior leaflet coupled with a pericardial posterior annuloplasty. This showed good coaptation **(A)**, laminar flow through the valve and excellent mobility of the leaflet **(B)** [Courtesy of Aswan Heart Center (AHC) database].

Rheumatic mitral valves are eminently repairable, especially in children (Figure 10) (85), with postoperative results sometimes outperforming balloon mitral valvuloplasty (86) and with re-intervention rates similar to those of MV replacement (87–89). Rheumatic mitral valve repair has the benefit of lower early and long-term mortality together with freedom from valve related complications. These benefits are not attributable to avoidance of anticoagulation alone, as the use of bioprosthetic valves in rheumatic valve replacement was associated with more valve related adverse events than valve repair or mechanical valve replacement in a recent report from Ethiopia (90). Valve repair should therefore be the primary goal in rheumatic heart disease, when feasible (88). However, patients should be carefully selected for mitral valve repair, particularly those with previous percutaneous transvenous mitral commissurotomy (91).

Yet despite recent evidence, the majority of patients with rheumatic mitral valve disease are still treated with valve replacement. This is due to the peculiar anatomical/pathological features of rheumatic valves (like fibrosis, retraction, calcific degeneration, and subvalvular apparatus involvement) unpredictable disease progression over time and historically poor outcomes of repair. Additionally, due to its rarity in high income countries, the majority of surgical expertise in valve repair has focused on degenerative and connective tissue disorders while rheumatic disease has not received similar attention.

To improve the adoption of mitral repair for rheumatic valve disease, techniques need to be made more reproducible and results followed up. This can be achieved by identifying selection criteria for repair candidates (91–93), standardizing a methodical approach to rheumatic mitral valve repair which can be taught and audited (88, 94, 95) and, importantly, strengthening local surgical programs in endemic areas, with expert valve repair surgeons providing technical guidance as well as mentorship on systematic follow-up and audit (90, 96). Collaboration of local surgical programs, through mutual site visits, workshops, or regional meetings, can have a significant effect on the wide adoption of rheumatic valve repair and the improvement of its techniques.

Surgical techniques for rheumatic aortic valve disease are still evolving. There is a need to evaluate current practice by following up large numbers of patients, focusing specifically on ventricular function and quality of life, which is often significantly impaired. The Ross procedure, where the diseased aortic valve is replaced with the patient's own pulmonary valve while a pulmonary allograft is used to replace the patient's own pulmonary valve, seems to be the best option for young patients as it is associated with reduced mortality, a positive impact on left ventricular function, better exercise tolerance and quality of life as well as having the benefit of avoiding anticoagulation. However, the number of patients which can be treated is limited by homograft availability (97).

A number of reports regarding surgery for rheumatic valve disease have been published recently, mostly from Asia. Fu and colleagues from Beijing, China have published their own experience (93), as well as a metanalysis (88), comparing outcomes between repair and replacement. They concluded that the repair group had better early mortality and less valve related events with similar reoperation rates. Kim et al. from Seol, Korea who reported their outcomes of 1171 patients undergoing surgery for rheumatic mitral valve disease found no difference in mortality between repair and replacement with significantly lower valve related complications in the repair group, again, without difference in the rate of reoperation. Waikittipong et al. published data on long-term outcomes after rheumatic mitral repair, reporting freedom from reoperation of 96, 90, and 82%, at 5, 10, and 15 years respectively (98). Krishnamurty et al. reported a 94% survival rate, 20 years after mitral valve repair in children with freedom from reoperation not statistically different from replacement but a rate of major adverse cardiac and cerebrovascular events (MACCE) that is significantly lower (87).

Taking a different point of view, Chen et al. (Taiwan) have performed propensity matching for 5086 adult patients who underwent surgery for rheumatic mitral valve disease with a mean follow up of 6 years could not find a superiority for mitral valve repair over replacement in terms of long-term outcomes. However, this paper compared 489 repairs and 4597 replacements. The same sentiment was shared with Russel et al. from Australia who found no difference between repair and replacement for rheumatic valves and no difference between surgery for rheumatic and non-rheumatic valves (99). Pillai et al. also looked at outcomes for concomitant aortic and mitral valve replacement and reported a survival rate of 95, 93, and 93% at of 1, 5, and 10 years, respectively with freedom from MACCE of 94, 89, and 85% (100). A collaborative study of a Canadian team visiting Ethiopia has looked at outcomes of surgery for rheumatic valve disease and found acceptable rates of major valve-related adverse events albeit significantly higher in bioprosthetic valves (90).

Lu and colleagues have published interesting articles on standardizing repair techniques for rheumatic mitral repair as well as their results in commissurotomy, as the main technique, for treating mitral stenosis. They have also proposed a grading system to identify features that make rheumatic mitral valves suitable for repair. They compared their mid-term results of 921 patients receiving repair (221) vs. replacement (700) and found increased incidence of heart failure in the replacement group as well as increased valve related complications and worse quality of life.

It remains evident that outcome of rheumatic mitral valve (MV) repair is excellent in terms of early and long-term survival as well as freedom from valve-related complications, commonly seen in rheumatic MV replacement (88). Until rheumatic valve disease is medically preventable the cardiac surgical community is responsible for improving the surgical treatment offered to these patients. This can be achieved by case selection and improving the adoption of valve repair as well as standardizing techniques, auditing outcomes and following results. This is exemplified by the regular workshops carried out by the Aswan Heart Centre which attracts cardiac surgeons from all over the region to discuss and exchange experience in rheumatic mitral valve repair as well as surgery for atrial fibrillation. The workshop discusses the current literature, the anatomical and physiological concepts of mitral valve pathology in health and disease and the various surgical techniques to repair these pathologies. It is augmented by presentations of recorded videos and live cases as well as wet lab sessions for surgeons in training (101).

**10. “Develop tissue-engineered valve substitutes, including percutaneous valves that are both affordable and simple to implant.”**

**Figure 11:**
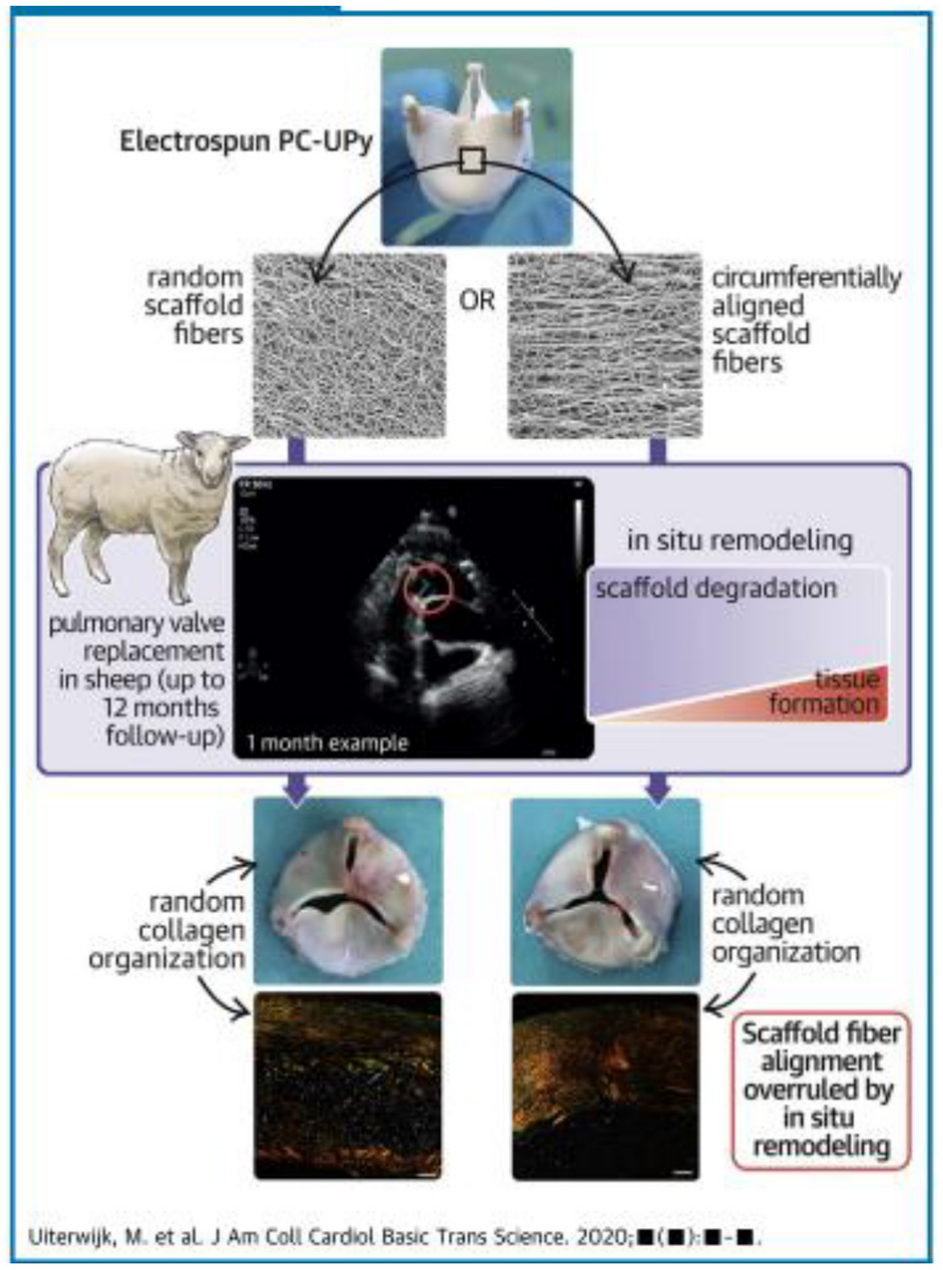
Concept of *in situ* tissue engineering heart valves: electrospun tri-leaflet valve scaffolds of ureidopyrimidinone-polycarbonate with either a random fiber alignment (rTEHV) or with fibers with a predominant alignment in the circumferential direction of the valve leaflet (aTEHV). Electrospun tubular conduits are sutured onto a crown-shaped polyether ether ketone supporting ring (outer diameter 20 mm, inner diameter 18 mm) to create a tri-leaflet valvular shape. The scaffold microstructure is analyzed via scanning electron microscopy (SEM), and fiber alignment is quantified. *In vitro* valve functionality is evaluated in a hydrodynamic pulsatile duplicator system under physiological pulmonary conditions and sterilized by gamma irradiation. Subsequently, surgical orthotopic pulmonary valve replacement is performed in female Swifter sheep (102).

The technology and concepts involved in tissue engineering of heart valves continue to evolve, with more concentration on *in situ* tissue engineering (Figure 11) (102–105).

**Figure 12:**
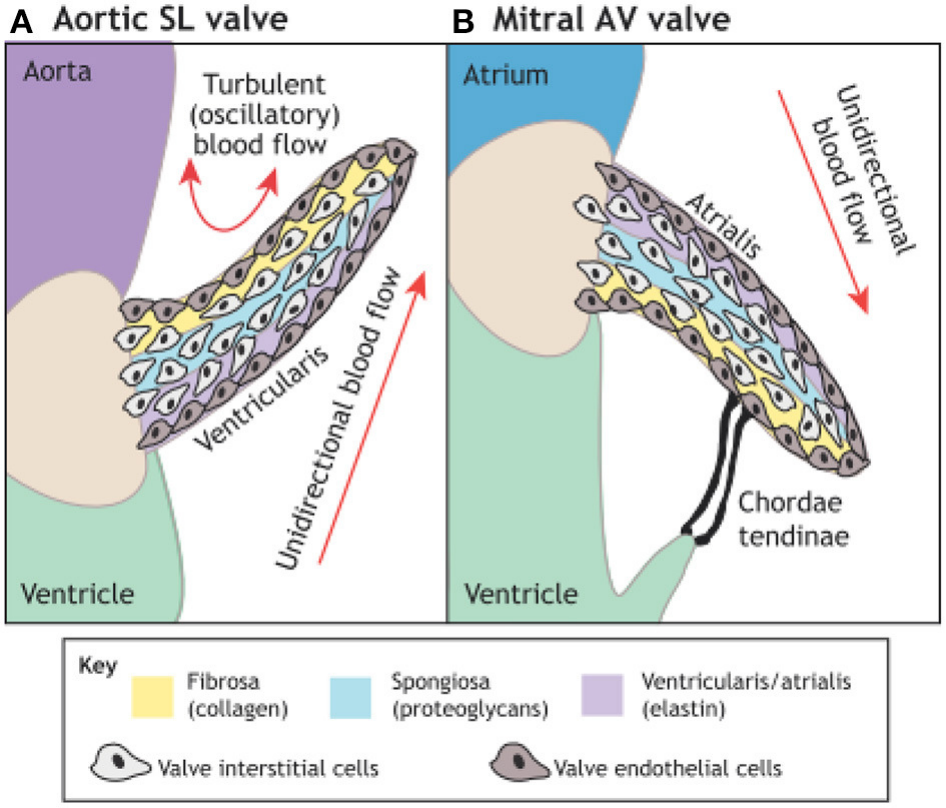
The microarchitecture of adult cardiac valves **(A,B)**. The stratified structure of aortic **(A)** and mitral **(B)** cardiac valves ensures unidirectional blood flow. Valve leaflets are composed of three layers of stratified extracellular matrix, including an elastin-rich ventricularis layer in the SL valves or the atrialis layer in the AV valves (purple), a proteoglycan-rich spongiosa layer (light blue) and a collagen-rich fibrosa layer (yellow), interspersed with valve interstitial cells (VICs) and sheathed in a monolayer of valve endothelial cells (VECs). The direction of pulsatile blood flow in relation to valve leaflets is indicated (red arrows). The microarchitecture of an aortic valve leaflet ensures blood moves from the ventricle to the aorta. The microarchitecture of a mitral valve leaflet is supported by the chordae tendineae, which ensure blood flow from the atrium to the ventricle. AV, atrioventricular; SL, semilunar (106).

**Figure 13:**
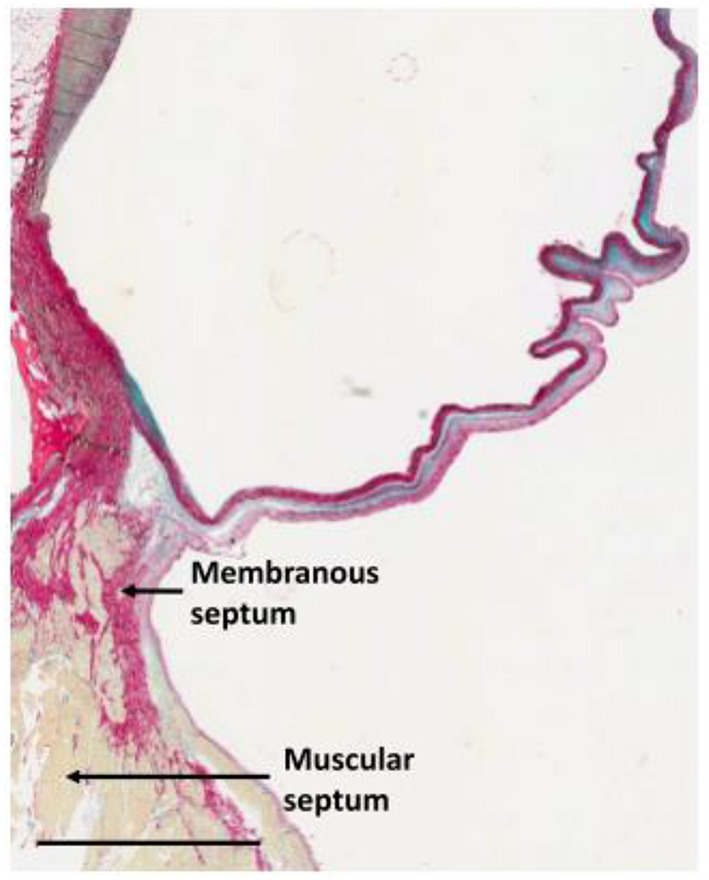
Section through the anterior half of the non-coronary sinus approaching the interventricular septum illustrating the concept of the trilammellar sliding hypothesis. Scale bar is 3 mm (107).

**Figure 14:**
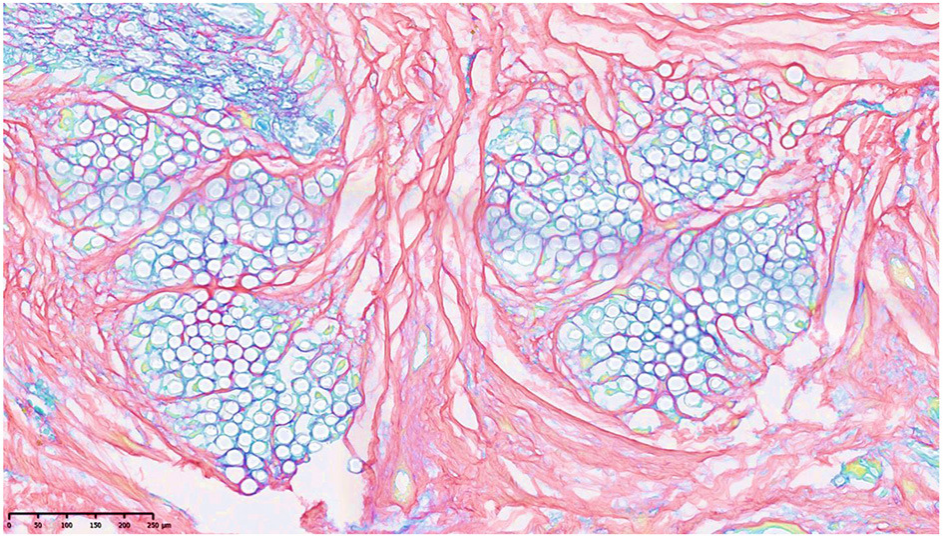
Forming of collagen between 2 membranes in response to the integrated jacket of PCL (gray circles) showing the possibility of manipulation of collagen in tissue engineering. The stain is picrosirius (108).

These techniques rely on understanding and exploiting the basic mechanisms involved in valve morphogenesis (Figure 12) (106) and normal valves (Figure 13) (107) with the use scaffolds capable of recruiting, housing, and instructing appropriate host cells and ECM (Figure 14) (108).

The absence of the desired control over the organization of regenerated tissue, as governed by the *in vivo* remodeling processes highlights the need for a more in-depth understanding of the long-term *in situ* remodeling processes in large animal models to improve predictability of outcome and outcome toward rational scaffold design and robust and safe clinical application (102).

Parallel progress in the design of affordable percutaneous tools for insertion of these valves is being made (109–111).

## Discussion

Although there has been considerable progress and much has been learned since the Cairo Accords were formulated, much remains to be done to enhance the global priority of ARF and RHD. There have been encouraging developments in the setting up and strengthening of registries and it is hoped that these will help fill major gaps in our understanding of the natural history of rheumatic heart disease and may help identify why RHD is so closely linked with socioeconomic development. Longitudinal studies are still needed to evaluate the clinical significance of asymptomatic RHD and to assess whether echocardiographic screening of populations for early disease could play a role in the control of RHD although the available data suggests that echocardiographic screening may find its place as a research tool. Although progress has been made on the genetics of rheumatogenic streptococcal strains as well as genetic determinants of susceptibility in patients, the work carried out underlines the complexity of the processes involved and the difficulties of translation into clinical practice. Encouraging progress, however, has been made in the development of polyvalent streptococcal vaccines but these will need large–scale funding for appropriate clinical trials.

While it is clear that secondary prophylaxis with penicillin continues to play a key role in disease control, current practice is still dependent on clinical protocols developed nearly 70 years ago. Better understanding of the pharmacokinetic and pharmacodynamic characteristics of penicillin and its possible alternatives are essential for improved strategies and regimens, especially in the context of pressures to reduce the use of antibiotics. Early diagnosis and improved diagnostic biomarkers are expected to impact on early detection and prognosis, while the potential value of anti-inflammatory and immunosuppressive therapy should be extensively studied for its effect on the treatment and prognosis of ARF. Because of the complexity of the disease and the involvement of several clinical and academic disciplines, collaboration between the different RHD initiatives is essential, with cross-communication between researchers and mechanisms for data exchange. The establishment of regional and global research networks should be promoted, to enhance the impact of the strategies. Finally, while surgery has always played a central role in RHD treatment, there is much potential for the development of new technology and surgical exchange programmes should be encouraged. One important area is the need to evaluate rheumatic valve repair and the development of improved tissue-engineered valve substitutes as the most effective treatment option for valve disease, especially in the young.

## Conclusion

This review emphasizes the need for continuing to pursue the target of eradicating RHD with vigor, and illustrates the value of applying a comprehensive systematic approach as articulated in the Cairo Accord.

## Author Contributions

SK and MY contributed to the conception and design of the study. SK wrote the first draft of the manuscript. SK, DP, and MY wrote the main manuscript. AA wrote sections of the manuscript. All authors contributed to manuscript revision, read, and approved the submitted version.

## Conflict of Interest

The authors declare that the research was conducted in the absence of any commercial or financial relationships that could be construed as a potential conflict of interest.

## Abbreviations

ARF: Acute Rheumatic Fever
RHD: Rheumatic Heart Disease
ARGI: Aswan Rheumatic Heart Disease Registry
DALYs: disability-adjusted life-years
LMICs: middle-income countries
GAS: group A streptococcus
GWAS: genome-wide association studies
IM: intramuscular
BPG: benzathine benzylpenicillin G.
